# Endoscopic Bridging Stent Placement Improves Bile Leaks After Hepatic Surgery

**DOI:** 10.3390/jcm14103381

**Published:** 2025-05-13

**Authors:** Taisuke Obata, Kazuyuki Matsumoto, Kei Harada, Nao Hattori, Ryosuke Sato, Akihiro Matsumi, Kazuya Miyamoto, Hiroyuki Terasawa, Yuki Fujii, Daisuke Uchida, Shigeru Horiguchi, Koichiro Tsutsumi, Motoyuki Otsuka

**Affiliations:** Department of Gastroenterology and Hepatology, Okayama University Hospital, 2-5-1 Shikata-cho, Okayama 700-8558, Japan; p47691mh@okayama-u.ac.jp (T.O.); pxcy7u17@okayama-u.ac.jp (K.H.); pqfn35i5@okayama-u.ac.jp (N.H.); p2r1565v@okayama-u.ac.jp (R.S.); pxbb7o3o@okayama-u.ac.jp (A.M.); p5vn01ag@okayama-u.ac.jp (K.M.); p0yo3av1@okayama-u.ac.jp (H.T.); pmug1j9r@okayama-u.ac.jp (Y.F.); uchida0309@okayama-u.ac.jp (D.U.); p4nc20ad@okayama-u.ac.jp (S.H.); tsutsumi@cc.okayama-u.ac.jp (K.T.); otsukamoto@okayama-u.ac.jp (M.O.)

**Keywords:** bile leak, endoscopic treatment, bridging

## Abstract

**Background**: Endoscopic treatment is one of the first-line treatments for bile leaks after hepatic surgery. However, detailed reports of endoscopic treatment for bile leaks after hepatic resection (HR) or liver transplantation (LT) are scarce. The outcomes of endoscopic treatment for bile leaks after hepatic surgery were examined, and factors related to successful treatment were identified. **Methods**: A total of 122 patients underwent endoscopic treatment for bile leaks after hepatic surgery. The diagnosis of a bile leak is based on the ISGLS criteria. The decision to perform endoscopic retrograde cholangiography (ERC) is made based on the amount of drainage output, laboratory data, clinical symptoms, and CT scan findings. In our study, the site of the bile leak was assessed using ERC. Endoscopic stents were placed to bridge across the bile leak site as much as possible. Otherwise, stents were placed near the leak site. Endoscopic stents were replaced every 2–3 months until an improvement in the bile leak was observed with or without biliary strictures. The outcomes of endoscopic treatment and the factors related to clinical success were evaluated. **Results**: Seventy-four patients with HR and forty-eight patients with LT were treated endoscopically. Technical and clinical success was achieved in 89% (109/122) and 82% (100/122) of patients, respectively. Three (2%) patients died from uncontrollable bile leaks. Bridging stent placement (*p* < 0.001), coexistent percutaneous drainage (*p* = 0.0025), and leak severity (*p* = 0.015) were identified as independent factors related to the clinical success of endoscopic treatment. During a median observation period of 1162 days after the achievement of clinical success, bile leak recurrence was observed in only three cases (3%). **Conclusions**: Endoscopic treatment is safe and effective for bile leaks after hepatic surgery. Bridging stent placement across the leak site is the most crucial factor for clinical success.

## 1. Introduction

The number of patients with biliary tract cancer (BTC) is increasing [1,2,3]. Hepatocellular carcinoma (HCC), despite its advances in antiviral therapy for hepatitis viruses, remains the 13th most diagnosed cancer worldwide and is the 7th leading cause of cancer-related deaths [4,5]. Hepatic resection (HR) is one of the curative treatments for these cancers, and about 10,000 patients undergo liver transplantation (LT) each year in the United States [6], aiming to obtain a complete cure for both benign and malignant liver diseases. A bile leak is one of the most common adverse events after hepatobiliary surgery, occurring in 4.5–18% of patients [7,8,9,10,11,12,13].

Infection with a bile leak is a well-known cause of sepsis and abscess formation, which can be fatal [14,15,16]. Reoperation is considered a highly invasive therapy; thus, percutaneous transhepatic biliary drainage (PTBD) is commonly performed to resolve bile leaks, but it has some limitations [17,18]. PTBD is challenging to perform under the continuous administration of anticoagulants or with uncontrolled ascites [19,20]. Moreover, quality of life (QOL) is impaired during the period when an external fistula tube is in place. Endoscopic treatment overcomes these disadvantages and allows stent placement within the body as an internal fistula [21].

Since the first initial report in the 1990s [22], clinical success rates of endoscopic retrograde cholangiography (ERC) for bile leaks after cholecystectomy have been frequently investigated, with reports ranging from 79 to 100% [22,23,24,25,26,27,28]. It is generally recommended to combine endoscopic sphincterotomy (EST) with stent placement in treatment and place the stent so that it bridges across the leak site for effective drainage [29,30,31,32]. Reports of endoscopic treatment for bile leaks after HR or LT are rare, with clinical success rates ranging from 64 to 89% [33,34,35,36]. Due to the anatomical complexity, ERC procedures after hepatic surgery for bile leaks are more challenging compared to after surgery for the common bile duct or cystic duct. Although the location of stents is an important factor related to the improvement of bile leaks [37], detailed investigations of this have not been conducted so far in patients after hepatic surgery.

In this study, the outcomes of ERC treatment for bile leaks after hepatic surgery were investigated, and the factors related to clinical success after endoscopic treatment were evaluated.

## 2. Materials and Methods

### 2.1. Patients

Between July 2004 and December 2022, 122 consecutive patients underwent initial endoscopic treatment for bile leaks after hepatobiliary surgery at Okayama University Hospital (Okayama, Japan). In all cases, external drain tubes were placed near the resection site during surgery, and the diagnosis of a bile leak was based on the ISGLS 2010 criteria [38]. The decision to perform ERCP was made based on the amount of drainage output, laboratory data, clinical symptoms, and CT scan findings. This study included the following patients: (1) those who had confirmed fluid retention on postoperative CT after liver transplantation or hepatic resection, accompanied by clinical symptoms such as fever, abdominal pain, and an abnormal blood test result; and (2) men and women aged 20 years or older. The following patients were excluded: (1) those who opted for conservative treatment only and (2) those with significant abnormalities in their vital signs, precluding endoscopic procedures. This study was approved by the Ethics Committee of Okayama University Hospital in accordance with the guidelines of the Declaration of Helsinki (approval number 2408–004, dated 31 May 2024).

### 2.2. Endoscopic Procedure

**Figure 1:**
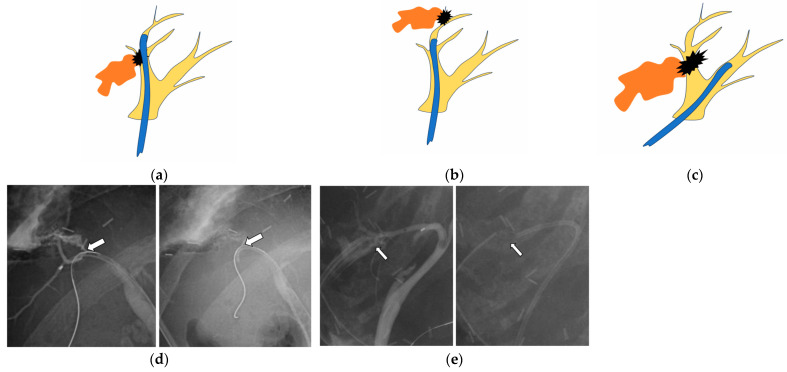
The stent is placed (**a**) bridging across the leak site, (**b**) distal to the leak site, or (**c**) at another bile duct near the bile leak site. (**a**–**c**) Yellow: bile duct branch, black: leak site, orange: leaked bile, blue: plastic stent. (**d**) The bile leak (arrow) is found at B6, and a stent is placed bridging across the leak site. (**e**) The leak (arrow) is found at B5, and a stent is placed distal to the leak site.

Endoscopic treatments were performed using duodenoscopes JF-260V, TJF-260V, and TJF-Q290V (Olympus, Tokyo, Japan). Patients were consciously sedated with intravenous sedative drugs. After biliary cannulation, cholangiography was performed. Bile leaks were recognized by the leakage of contrast medium from any part of the biliary tract, and endoscopic stent placement using a plastic stent (PS) was performed. Basically, a PS was placed to bridge the leak site. That is, the proximal end of the PS was placed in the biliary tract upstream of the leak site. If the proximal bile duct at the leak site was narrow or not identified, or the PS did not stabilize well due to the angle or bifurcation of the bile duct, the PS was placed distal to the leak site or in another nearby bile duct (Figure 1). If endoscopic stenting was not successful due to a severe bile leak or stricture, PTBD or re-operation was considered a form of salvage treatment.

Technical success was defined as successful biliary stent placement at the intended site. Clinical success was defined as an improvement in clinical symptoms (fever, abdominal pain, etc.) after treatment. An improvement in a bile leak was defined as the disappearance of the bile leak on ERC after stent placement. The severity of bile leaks was classified into two groups, according to the report by Sandha et al. [26]. In low-grade cases, a bile leak was observed after filling the intrahepatic bile duct with a contrast agent, and in high-grade cases, it was observed before filling the intrahepatic bile duct with a contrast agent (Supplementary Figure S1). Adverse events due to endoscopic procedures were evaluated according to the ASGE 2010 guideline [39].

### 2.3. Follow-Up After Stent Placement by ERC

After stent placement, ERC was performed 2–3 months later. If the bile leak was resolved on ERC, the PS was removed. If the bile leak did not improve or a bile duct stricture remained regardless of the improvement in the bile leak on ERC, PS placement was repeated every 2–3 months until an improvement in the bile leak or bile duct stricture was observed. For patients who achieved stent-free status, ERC was not performed unless there was a recurrence of clinical symptoms. Patients were followed up with blood tests and/or imaging examinations at least every six months. A typical case is presented in Supplementary Figure S2.

### 2.4. Statistical Analysis

Continuous variables were presented as median and range or interquartile range (IQR) values. Comparisons of continuous variables were made using the Wilcoxon signed-rank test. Comparisons of dichotomous variables were performed using Fisher’s exact test. Logistic regression analysis was performed to identify important factors affecting the outcome of endoscopic procedures for postoperative bile leaks. *p* < 0.05 was considered significant. JMP 16 statistical software (SAS Institute Inc., Cary, NC, USA) was used for all statistical analyses.

## 3. Results

### 3.1. Patients’ Characteristics

The patients’ characteristics are shown in Table 1. The total number of cases was 122. Background diseases included hepatocellular carcinoma (*n* = 51), cholangiocarcinoma (*n* = 9), other cancers/tumors (including metastatic liver tumors) (*n* = 14), liver failure due to liver cirrhosis (*n* = 40), transplant donors (*n* = 5), and three other cases. The median interval from surgery to the first ERCP was 41 days (IQR 22–85 days), with surgical procedures including left or right lobectomy (including extended resection) (*n* = 38), segmental resection (*n* = 36), and LT (*n* = 48). LT consisted of living donor liver transplantation (LDLT) (*n* = 47) and deceased donor liver transplantation (DDLT) (*n* = 1). At the time of the initial ERCP, 89 patients (72%) had an existing external drainage tube in place. Of these, 68 patients retained the surgical drain placed during surgery, whereas 21 patients underwent additional percutaneous transhepatic biliary drainage under abdominal ultrasound guidance.

### 3.2. Results of ERCP

**Figure 2:**
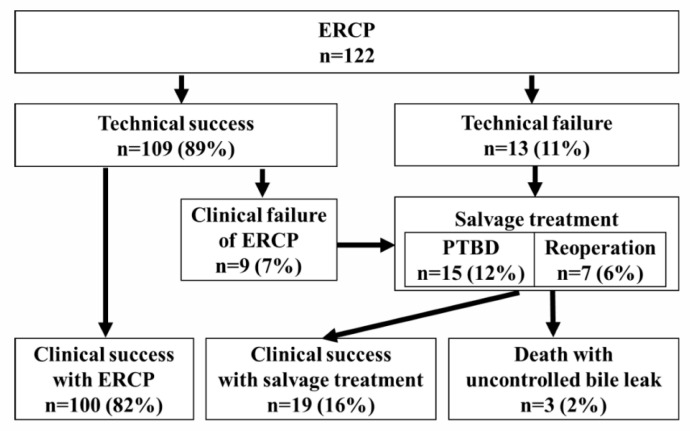
A flow diagram of bile leak management from the first ERCP to clinical success. Technical success: successful biliary stent placement at the intended site. Clinical success: improvement in clinical symptoms.

Table 2 shows the results of the ERCP. Bile leak sites in HR cases included a common hepatic duct in 2 cases, a hilar bile duct in 54 cases, and a peripheral bile duct in 18 cases. In LT cases, bile leaks were observed in 44 cases at the bile duct anastomosis and in 4 cases at the residual cystic duct. EST was performed in 65 cases (53%), including those performed previously. A PS was placed to bridge the leak site in 82 cases (67%), at the distal site of the bile leak in 12 cases, and at another nearby bile duct in 15 cases. Technical success was achieved in 109 cases (89%). In the 13 cases (11%) with technical failure, the guide wire or stent could not be delivered proximally to the bile leak site (Supplemental Table S1). Of the cases with technical success, 100 (90%) had clinical success. A total of 22 cases (with 13 technical and 9 clinical failures) included 15 cases of PTBD and 7 cases of reoperation as forms of salvage treatment. The improvement in bile leak symptoms was achieved in 19 cases, which was considered clinical success with salvage treatment. Three cases died due to uncontrolled bile leaks (Figure 2).

Adverse events during the initial ERCP procedure for bile duct leaks were observed in nine cases (7%). There were seven cases of post-ERCP pancreatitis (6%), two cases of cholangitis (2%), and one case of cholangitis with a liver abscess (1%); all cases improved with conservative treatment.

### 3.3. Long-Term Outcomes

**Figure 3:**
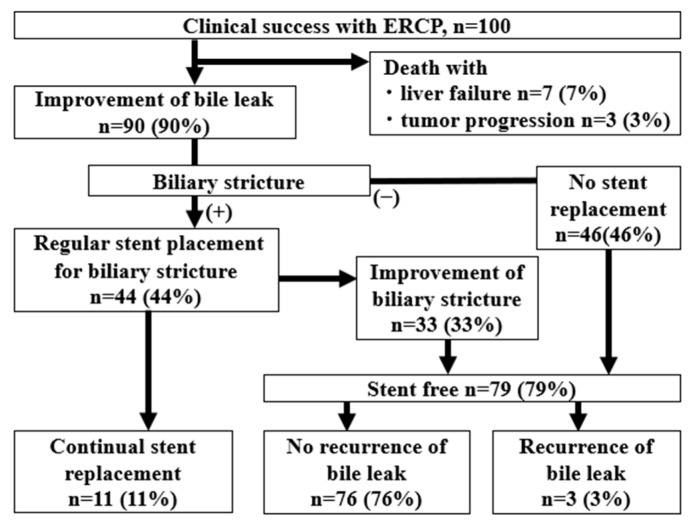
A flow diagram showing the outcomes from clinical success. After clinical success, the bile leak disappeared in 90% of cases, of which 79% of cases were at least temporarily stent-free, and 11% had persistent stenosis requiring regular stent replacement.

Of the 100 cases that achieved clinical success with ERCP, 90 cases (90%) had improved bile leaks. The median day until the improvement of the bile leak was 122 days (IQR 82–200 days). Ten patients died from liver failure after the bile leak was controlled during the follow-up period. Of the 90 patients with an improvement in bile leaks, 46 had no bile duct strictures and became stent-free, and 44 patients had remaining bile duct strictures that required repeat ERCP. Of them, 33 patients had improved bile duct strictures at a median of 189 days (IQR 91–367 days), whereas 11 patients still had significant strictures and required stent replacement (with a median observation period of 527 days (IQR 150–1134 days). After the achievement of stent-free status, bile leaks recurred in three patients, and repeat ERCP was performed (112, 180, and 1352 days after stent removal). The remaining 76 stent-free patients had no recurrent bile leaks during the median observation period of 1162 days (IQR 350–1781 days) (Figure 3).

### 3.4. Factors Related to Clinical Success of Endoscopic Treatments

Factors related to the clinical success of endoscopy were evaluated. Conducting univariate analysis showed that leak severity (*p* = 0.019), a leak-bridging stent (*p* = 0.001), and coexisting external fistulas (*p* = 0.010) were significant. For multivariate analysis, a bridging stent (OR, 12.2; 95% CI, 3.49–42.5; *p* < 0.001), coexisting external fistulas (OR, 7.10; 95%CI, 1.99–25.3; *p* = 0.0025), and leak severity (OR, 0.21; 95% CI, 0.06–0.74; *p* = 0.015) were also found to be independent factors related to clinical success (Table 3).

## 4. Discussion

In this study, endoscopic treatment outcomes for bile leaks were evaluated in 122 patients after hepatic surgery. The technical and clinical success rates were 89% and 82%, respectively. Three patients died due to uncontrollable bile leaks. After the achievement of clinical success, only three patients had recurrent bile leaks with a median follow-up of 1162 days. Bridging stent placement was related to clinical success.

PTBD has traditionally been used to treat bile leaks after hepatic surgery [17,18,19,20]. Whereas external fistulas offer the advantage of allowing the visual assessment of drainage, the performance of contrast studies involving flushing the lumen shows that prolonged placement significantly reduces patients’ QOL. Recently, the effectiveness of endoscopic stenting for bile leaks has been recognized [26,28,40,41]. However, only a few reports have examined in detail how to place stents and how to intervene in the bile ducts. In the present study, bridging stent placement across the leak site was positively related to clinical success. Regarding the placement of stents, positioning them distally or in nearby branches supports this pressure gradient. In contrast, promoting the closure of the fistula is also considered crucial in treating bile leaks. Bridging stent placement not only reduces bile duct pressure and decreases bile leaks, but it also facilitates fistula closure by reducing bile contact with the fistula site [29,30]. Thus, in ERC for bile leaks after cholecystectomy, it is considered most effective to place a stent bridging the leak site [24,29], and the present study suggests that this is also true for bile leaks after hepatic surgery.

EST is believed to promote the healing of bile leaks by reducing the pressure gradient across the sphincter of Oddi through an incision, thereby increasing bile flow into the duodenum [42,43,44,45]. In the present study, including cases that had already undergone previous examinations, EST was performed in 65 of 122 cases (53%). The clinical success rates were 80% (52/65) in the group with EST and 82% (41/50) in the group without EST, showing no significant difference. There were two reasons for this result. First, all stents were placed across the papilla, so the pressure in the biliary duct decreased with or without EST. Second, there may have been differences in the background factors that determined whether EST was performed. In cases of hepatic resection (HR), when there was concern about the recurrence of hepatocellular carcinoma, EST tended to be avoided (HR: 36/73; LT: 29/49; *p* = 0.35).

Regarding the size and number of stents placed, previous reports did not find differences between large and small-diameter stents [29,46]. In the present study, stent diameter ≥7 Fr and multiple stents were not significantly associated with clinical success. We previously reported that the severity of bile leaks was negatively related to the improvement of bile leaks [47]. The severity here was defined as mild if the leak was seen after peripheral cholangiography was performed and severe if the leak was seen from the early stages of cholangiography. The present results were the same in that less clinical improvement and bile leak improvement were seen in the severe bile leak group.

In the endoscopic treatment of bile leaks, while demonstrating the effectiveness of stent placement primarily focuses on bridging, there has been a renewed recognition of the efficacy of external fistula creation. In the present study, a surgical drain and/or PTBD was used in 89 cases (72%) during endoscopic treatment, and ERCP was performed in cases that did not show improvement. PTBD enabled biliary pressure reduction, infection control, irrigation, and the confirmation of biliary tract anatomy through contrast imaging. PTBD placement affected technical success, clinical success, and improvements in bile leaks. In refractory cases, approaching both sides is considered necessary.

This study had limitations. This was a retrospective, single-center analysis. However, reports of endoscopic treatment for bile leaks after hepatic surgery have been scarce. This report included a large number of cases and provided long-term follow-up after treatment. Also, although some reports have described the effectiveness of self-expanding metallic stents (SEMSs) for patients with bile leaks after pancreaticobiliary surgery [45,48], Japanese health insurance does not allow the use of SEMSs for benign diseases. Nevertheless, placing SEMSs in a narrow peripheral bile duct is controversial because of the possible obstruction and excessive dilatation of bile duct branches. Finally, this study does not clarify the total number of patients who developed a bile leak among those who underwent hepatic surgery during the study period, nor the proportion of those who received endoscopic treatment.

## 5. Conclusions

Endoscopic treatment is safe and effective for bile leaks after hepatic surgery. Bridging stent placement across the leak site should be attempted first.

## Figures and Tables

**Table 1:** Patients’ characteristics.

Number of Patients	122	Surgery Type, *n* (%)	
Sex, male, *n* (%)	89 (73)	LDLT	48 (39)
Median age, years (range)	60 (21–85)	Right lobe graft	30 (25)
Diagnosis, *n* (%)		Left lobe graft	14 (11)
Hepatocellular carcinoma	51 (42)	Posterior segment graft	4 (3)
Cholangiocarcinoma	9 (7)	DDLT	1 (1)
Other cancers/metastatic cancer	14 (11)	Total liver graft	1 (1)
Liver failure due to liver cirrhosis	40 (33)	Hepatic resection	73 (60)
Transplant donors	5 (4)	Right lobe resection	13 (10)
Other benign disease	3 (2)	Left lobe resection	24 (20)
Interval between surgery and	41 (3–1716)	Segment resection	36 (30)
First ERCP, median, days (range)		Bile leak grade (ISGLS 2010 [38]), *n* (%)	
With external fistula at the initial ERCP	89 (72%)	Grade B Grade C	115 (94) 7 (6)

LDLT, living donor liver transplantation; DDLT, deceased donor liver transplantation.

**Table 2:** ERCP results.

Bile Leak Location, *n*		Plastic Stent Placement, *n* (%)	
HR		Bridging the leak site	82 (67)
Hilar duct	54	Distal of the leak site	12 (9)
Peripheral duct	18	Another bile duct	15 (12)
CHD	6	Median number of stents, *n* (range)	1 (1–3)
LT		Maximum stent diameter	7 (5–10)
Anastomosis	44	Median, Fr (range)	
Cystic duct remnant	4	Clinical success, *n* (%)	100 (82)
Biliary stricture, *n* (%)	78 (62)	Salvage treatment, *n* (%)	
Bile leak severity, *n*		PTBD	15 (12)
Low grade	61	Reoperation	7 (6)
High grade	61	Adverse events, *n* (%)	
Endoscopic sphincterotomy, *n* (%)	65 (53)	Pancreatitis	7 (6)
Technical success, *n* (%)	109 (89)	Cholangitis	2 (2)
		Abscess	1 (1)

HR, hepatic resection; CHD, common hepatic duct; LT, liver transplantation.

**Table 3:** Factors related to clinical success of endoscopic treatments.

Variable		Clinical Success		Univariate		Multivariate	
		***n***	**%**	**OR (95%CI)**	***p* Value**	**OR (95%CI)**	***p* Value**
Sex, male		72/89	81	0.75 (0.25–2.25)	0.79		
Age, <median(60 y.o.)		48/57	84	1.33 (0.52–3.40)	0.64		
Surgery type	LT	38/49	78	1.63 (0.64–4.13)	0.34		
	HR	62/73	85				
Days from surgery to ERCP,		53/60	88	2.42 (0.090–6.43)	0.1	1.91 (0.57–6.43)	0.30
<Median (41 days)							
Leak location	LT, anastomosis	35/45	78	1.17 (0.11–12.5)	0.99		
	HR, hilar duct	44/55	80	0.80 (0.20–3.26)	0.99		
Target lobe	Right lobe	72/84	86	2.22 (0.86–5.74)	0.12		
Biliary stricture	(+)	62/77	81	0.65 (0.23–1.83)	0.47		
Leak severity	High grade	44/61	73	0.23 (0.08–0.68)	0.0085	0.21 (0.06–0.74)	0.015
Sphincterotomy	(+)	52/65	80	0.75 (0.29–1.91)	0.64		
Leak-bridging stenting	(+)	77/82	84	8.93 (3.13–25.4)	<0.001	12.2 (3.49–42.5)	<0.001
Maximum Stent diameter	≥7 Fr	38/41	93	1.90 (0.48–7.46)	0.53		
Multiple stents	(+)	18/18	100	N.A.	0.35		
With external fistula	(+)	79/88	90	5.43 (2.04–14.4)	0.0010	7.10 (1.99–25.3)	0.0025

OR, odds ratio; CI, confidential interval; LT, liver transplantation; HR, hepatic resection; N.A., not available.

## Data Availability

The original contributions presented in this study are included in the article/Supplementary Material. Further inquiries can be directed to the corresponding authors.

## Supplementary Materials

The following supporting information can be downloaded at https://www.mdpi.com/article/10.3390/jcm14103381/s1. Figure S1: Severity of bile leaks; Figure S2: Case presentation. Table S1. Patients’ characteristics and technical and clinical failure.

## Abbreviations

The following abbreviations are used in this manuscript:

ASGE	American Society for Gastrointestinal Endoscopy
BTC	Biliary tract cancer
CT	Computed tomography
DDLT	Deceased donor living transplantation
ERC	Endoscopic retrograde cholangiography
ERCP	Endoscopic retrograde cholangiopancreatography
EST	Endoscopic sphincterotomy
HCC	Hepatocellular carcinoma
HR	Hepatic resection
IQR	Interquartile range
LDLT	Living donor liver transplantation
LT	Liver transplantation
PS	Plastic stent
PTBD	Percutaneous transhepatic biliary drainage
QOL	Quality of life
SEMS	Self-expanding metallic stents
