# Supplementary material for: Endoscopic Bridging Stent Placement Improves Bile Leaks After Hepatic Surgery
Source: J Clin Med. 2025 May 13;14(10):3381. doi: 10.3390/jcm14103381 (PMC12112124; doi:10.3390/jcm14103381)
Supplement: Supplementary file 1 [file jcm-14-03381-s001.zip › jcm-3589602-supplementary.pdf]

Supplementary

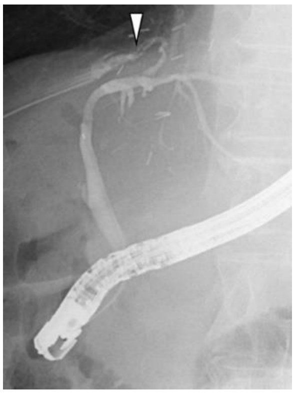

a

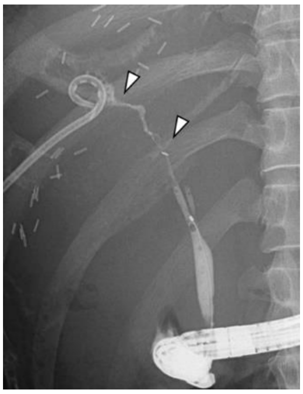

b

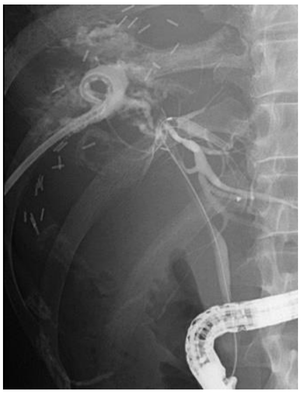

c

Supplemental Figure S1. Severity of bile leaks

A: A low-grade case. A bile leak is observed after filling of the intrahepatic bile duct with contrast agent (arrowhead) (a).

B: A high-grade case. A bile leak is observed before filling of the intrahepatic bile duct

with contrast agent (arrowhead). (b)Before filling with contrast agent. (c)After filling with contrast agent.

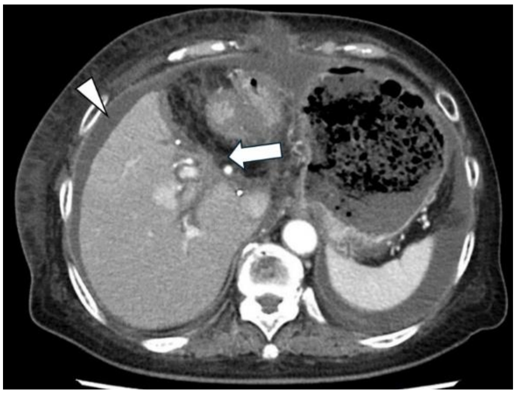

A

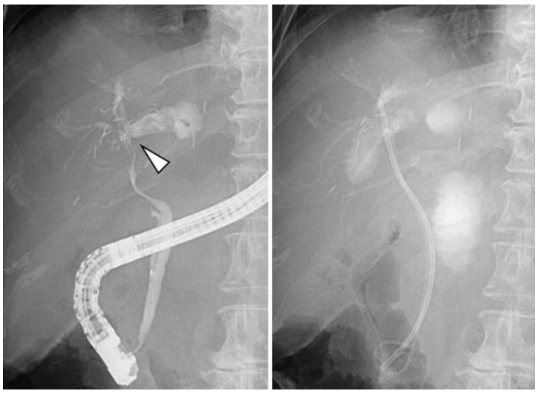

B

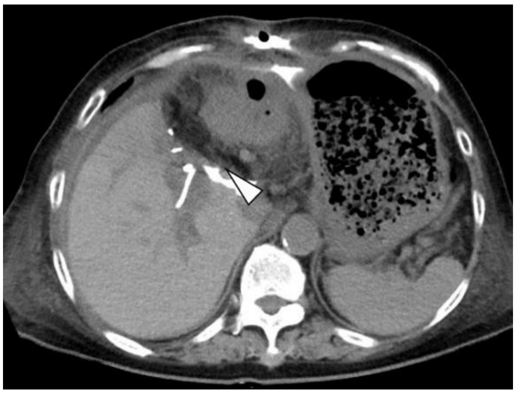

C

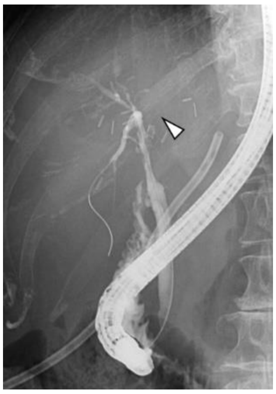

d

Supplemental Figure S2. Case presentation

A woman in her 70s underwent a left lobectomy for hepatocellular carcinoma. On postoperative day 45, she presented with a fever. CT showed fluid accumulation around the cut surface of the liver (arrow) and a small amount of ascites on the liver surface (arrowhead) (a). Suspecting a bile leak, ERC was performed. Cholangiography showed a high-grade leak from the hilar duct (arrowhead), and a bridging 7-Fr plastic stent was placed (b). The fever and inflammation subsided within a week. Subsequently, a PTBD was placed for fluid accumulation, but no new leaks were observed. Three months later, CT shows improvement of fluid accumulation (c). A follow-up ERC confirms the disappearance of the leak, and the stent is removed (d).

Supplemental Table S1. Patients' characteristics and technical and clinical failure

**Age (y)**	**Sex**	**Diagnosis**	**Surgery/ Procedure**		**Leak**	**Initial treatment**	**Cause of TF**	**Bridge stenting**	**Salvage treatment**	**Clinical success after salvage treatment**	**Outcomes**
**60s**	M	HCC	HR	Right lobe resection	HG	TF	GW Seeking		PTBD	Success	Death, other reason
**60s**	M	HCC	HR	Right lobe resection	LG	TF	Stent placement		PTBD	Success	Stent-free
**60s**	M	MLT	HR	Left lobe resection	HG	TF	GW Seeking		Reoperation	Success	Death, other reason
**70s**	M	HCC	HR	Left lobe resection	HG	TF	GW Seeking		PTBD	Success	Stent-free
**70s**	M	CCA	HR	Left lobe resection	HG	TF	Stent placement		PTBD	Success	Stent replacement
**80s**	F	CCA	HR	Left lobe resection	LG	TF	Stent placement		PTBD	Success	Stent-free
**60s**	M	HCC	HR	Segmentectomy	HG	TF	Stent placement		PTBD	Success	Death, other reason
**60s**	M	HCC	HR	Segmentectomy	LG	TF	Stent placement		PTBD	Success	Stent-free
**50s**	M	LF	LT	Right lobe graft	HG	TF	Stent placement		PTBD	Success	Stent-free
**40s**	M	LF	LT	Right lobe graft	HG	TF	GW Seeking		Reoperation	**Failure**	**Death, uncontrolled bile leak**
**60s**	F	LF	LT	Right lobe graft	HG	TF	GW Seeking		Reoperation	Success	Stent-free
**50s**	M	LF	LT	Left lobe graft	HG	TF	GW Seeking		Reoperation	Success	Death, other reason
**30s**	M	LF	LT	Left lobe graft	HG	TF	GW Seeking		PTBD	Success	Death, other reason
**70s**	M	HCC	HR	Segmentectomy	HG	CF		No	Reoperation	Success	Stent-free
**60s**	M	HCC	HR	Segmentectomy	LG	CF		No	PTBD	Success	Stent replacement
**70s**	M	MLT	HR	Segmentectomy	LG	CF		No	PTBD	Success	Stent-free
**50s**	M	HCC	LT	Right lobe graft	HG	CF		Yes	Reoperation	Success	Lost to follow-up
**50s**	F	HCC	LT	Right lobe graft	HG	CF		Yes	PTBD	**Failure**	**Death, uncontrolled bile leak**
**50s**	F	LF	LT	Right lobe graft	HG	CF		No	PTBD	Success	Stent-free
**50s**	M	LF	LT	Right lobe graft	HG	CF		Yes	Reoperation	Success	Stent-free
**60s**	F	LF	LT	Left lobe graft	HG	CF		Yes	Reoperation	Success	Stent-free
**30s**	M	LF	LT	Left lobe graft	HG	CF		No	PTBD	**Failure**	**Death, uncontrolled bile leak**

HCC, hepatocellular carcinoma; MLT, metastatic liver tumor; CCA, cholangiocarcinoma; LF, liver failure; HR, hepatic resection; LT, liver transplantation; HG, high grade; LG, low grade; TF, technical failure; CF, clinical failure; GW, guide wire; PTBD, percutaneous transhepatic biliary drainage
